# Life Course Approach in Social Epidemiology: An Overview, Application and Future Implications

**DOI:** 10.2188/jea.JE20140045

**Published:** 2014-09-05

**Authors:** Noriko Cable

**Affiliations:** Research Department of Epidemiology and Public Health University College London, London UK; Research Department of Epidemiology and Public Health, University College London

**Keywords:** life course approach, social gradients in health, social epidemiology

## Abstract

The application of the life course approach to social epidemiology has helped epidemiologists theoretically examine social gradients in population health. Longitudinal data with rich contextual information collected repeatedly and advanced statistical approaches have made this challenging task easier. This review paper provides an overview of the life course approach in epidemiology, its research application, and future challenges. In summary, a systematic approach to methods, including theoretically guided measurement of socioeconomic position, would assist researchers in gathering evidence for reducing social gradients in health, and collaboration across individual disciplines will make this task achievable.

## Abstract (ja)

ライフコースアプローチの社会疫学の分野への適用は、社会格差が国民の健康にどう影響を及ぼすかを理論的に研究するために役立っている。繰り返し収集された文脈的要因情報に富む縦断データや高度な統計手法によりライフコースアプローチによる社会格差の理論的な解明の困難さが軽減されていると言えよう。

本総説は、疫学におけるライフコースアプローチの概要、適用、及び将来に向けての課題を述べたものである。疫学研究者がライフコースアプローチによる健康格差縮小への確たる指標を打ち出すには 、理論的に裏付けられた社会経済地位測定因子も含め、体系的な考えに基づいた研究手法の適用、ならびに研究分野の枠を越えた各研究者の協力が望まれる。

## INTRODUCTION

Social epidemiologists are applying the life course approach in an effort to understand the longitudinal effects of socioeconomic adversities to which people are exposed at various developmental stages—especially in early years—on health. The life course approach, which involves studying life histories and trajectories, is not new to other disciplines.^1^ This approach has been receiving growing interest from researchers, owing to the availability of longitudinal and complex datasets and advances in statistical techniques. Application of this approach in social epidemiology has contributed to growing intellectual debates on the mechanisms of health inequalities over life time, which involves rich social contextual factors at both macro and micro levels.

It has long been a tradition in social epidemiology to examine health inequalities in relation to material, behavioural, and psychosocial pathways.^2^^,^^3^ Linking these pathways together, researchers have been attempting to identify biological plausibility of social gradients in health.^3^ As emphasised in the biological programming hypotheses, the effects of early life, beginning in utero, on health in later life are unarguably evident.^4^ This understanding of the significance of early life seems to have prompted the emergence of life course epidemiology, which broadly shares the aim of social epidemiology: to understand health inequalities through the three fundamental causal pathways (material, behavioural, and psychosocial). Life course epidemiology, however, places additional emphasis on time elements,^5^ including timing and a continuous sense of time (ie life-time). The purpose of this review is to illustrate the concepts and research application of life course epidemiology and to discuss the challenges that life course epidemiologists are facing and possible future directions for life course research.

## COMMON LIFE COURSE MODELS

Originally, two main conceptual models were identified to illustrate longitudinal associations between exposures to risk and health outcomes: the critical period model and the accumulation of risk model.^6^^–^^8^ Guided by Barker’s biological programming hypothesis, the emphasis of the critical period model is on the critical timing of exposure to risk factors that are likely to cause irreversible effects on health outcomes.^5^^,^^8^ Exposure to risk factors may occur in later life, but it is also possible to have modifying factors following the first exposure to risk factors.

The accumulation of risk model focuses instead on the patterns of exposures to multiple risk factors, which may be either independent or correlated (ie clustering or triggering). This model has been further divided into four models: (1) independent, (2) clustering, (3) chain of risk, and (4) trigger effect or stepping stone.^5^ Detailed descriptions of each type of risk accumulation model are reported elsewhere.^5^^,^^8^ Social mobility, defined as movements of social position from family of origin to adulthood (inter-generational) or within adulthood (intra-generational),^9^ can be incorporated into the risk accumulation model; specifically, the chain of risk model uses social mobility if individuals are repeatedly exposed to the same socially advantaged or disadvantaged position.^2^ This type of modeling is a crude way of estimating the effect of changes in social position on health throughout the course of an individual’s life, disregarding the patterns of such movement.

In regard to social mobility, the patterns of mobility (either upward, downward, or stable) were found to be equally significant in relation to health in later life, as well as timing or accumulation of exposure to social position.^9^ It has often been noted that the health of individuals who moved to an advantaged social position from a disadvantaged one (upward) appeared to be better compared to that of those who remained in the disadvantaged position.^10^ However, the health of these upwardly mobile individuals is likely to be worse than the health of those who remained in an advantaged position. Existing studies have attempted to reveal the complex mechanism of health inequalities across the life course through traditional social epidemiological pathways, with findings discussed in the next section. The social contextual factors associated with social mobility are worth further exploring theoretically and empirically.

## APPLICATION OF THE LIFE COURSE APPROACH TO QUANTITATIVE EPIDEMIOLOGICAL STUDIES

Many of the epidemiological studies that have examined social inequalities in population health have aimed to identify the role of the material, behavioural, and psychosocial pathways in relation to socially patterned health. Ploubidis et al fit all three pathways into their model to show the role of each in relation to the association between socioeconomic position and health and well-being of older adults in England.^11^

As stated in the introduction, the life course approach in social epidemiology incorporates those three causal pathways, which feed into social gradients in health, with particular attention focused on exposure in early years. Studies taking the life course approach use exposure in early childhood and examine the association between such exposure and outcomes at later life stages, taking care to account for mediators or moderators to ascertain longitudinal associations between social disadvantages and health across the life course. Using information obtained from British residents born in 1958 or 1970, Schoon, Bartley, and Sacker^12^ applied a path model to examine how a father’s occupation—an indicator of individual social position—in a child’s early years related to that child’s psychological health and social position in adulthood via psychosocial and material factors, which were repeatedly collected during childhood. Their findings showed direct paths from material risk factors in earlier childhood to psychosocial risk factors in later childhood, which in turn contributed to the level of social prestige and psychological health in adulthood. They also found that multiple exposures to the same risk factor were detrimental to these adult outcomes.

One way to test the critical/sensitive period and accumulation effect models is to rely on decomposition of correlational associations between exposures, mediators, and outcomes. As an example, Ploubidis et al^13^ examined the paths between recalled socioeconomic position and health during childhood and socioeconomic position and physical health in older age among independently living older adults aged 50 or over in England. They theorized that the *critical period* effect of childhood socioeconomic position on later life health outcomes could be identified based on the presence of a direct effect of this factor on the health outcome. An effect of *chains of risk* on later life health outcomes was tested by assessing the statistical significance of the indirect paths from childhood socioeconomic position to later life outcomes via the pathway from later life socioeconomic position. *Independent accumulation* effects from the exposure to socioeconomic position in early and later life were indicated by significance in the sum of direct effects from these factors on later life health outcomes. On stratification by gender and age groups, Ploubidis et al found that the critical role of childhood socioeconomic position was apparent in the group over 75 years of age.

Early socioeconomic status had a different effect on mortality in the study by Murray et al.^14^ They simultaneously fitted the critical period, accumulation, and social mobility models to examine the associations between life course socioeconomic position and various cardiovascular risk factors using the data from participants born in Great Britain in 1946. In their findings, the accumulation model predicted the association between socioeconomic position across the life course and cardiovascular risks in later life in women, whereas in men the outcomes were better predicted by early socioeconomic position. Mishra et al^15^ also detected gender-specific patterns predicting mortality in these models, although their findings were not consistent with the study by Murray et al.^14^

Kuh and Ben-Shlomo^5^ predicted that life course models to explain social determinants of health would include multiple contexts such as individual, family (ie cross-generational), environmental, and society-level factors as well as the social-biological pathway (ie embodiment). This view of the interaction of macro-level factors across generations has also been adopted by others.^2^^,^^3^^,^^8^

## FUTURE CHALLENGES

### Advantages and shortcomings of longitudinal data

Quantitative epidemiological studies, showing complex patterns of associations between socioeconomic position and health across the life course, depend on large longitudinal studies repeatedly collecting information since the birth of the participants (ie birth cohort studies). In Great Britain, established birth cohort study data, collected from residents in 1946,^16^ 1958,^17^ 1970,^18^ and 2000/1^19^ are available for use. Some information, especially on socioeconomic status and health, has been collected repeatedly.^16^^–^^19^

Having information collected from different birth cohorts is an advantage when assessing a period effect on outcomes. For example, Lacey et al^20^ tested the presence of ‘reduced effect’ on adult psychological health due to parental divorce occurring during childhood among British birth cohort study participants born in 1958 and 1970. It was possible to test their hypothesis, given the fact that parental separation was more common in 1970 than in 1958. These birth cohort studies are also useful in validating the effect of exposures on outcomes, such as the effect of breastfeeding on psychological health^21^ and social mobility.^22^

The availability of rich contextual data collected at various life stages is an advantage of a birth cohort study; however, missing information due to attrition is a major disadvantage in using such data. In the Millennium Cohort Study, data from close to 19 000 children were collected at baseline,^23^ and new eligible contacts were added to the existing cases; however, about 3000 cases were lost at the second wave, mostly due to refusal. One way to fill in the missing information is to use information from other data sources such as administrative data.^23^ Most of the parents of cohort children in the Millennium Cohort Study gave their consent for the data from birth registrations and maternity hospital records to be used; however, around 2200 fewer consents were obtained to link the data with the maternity hospital records than to link with birth registration (around 16 000).

Hockley et al^24^ reported that using these linked administrative data allowed them to complete the necessary information. Moreover, these data allowed researchers to validate information provided by mothers of the cohort children.^24^^,^^25^ For example, maternal recall on child’s birth weight was highly accurate (92% overall), though mothers from socioeconomically disadvantaged or ethnic minority groups were more likely to recall it inaccurately than those who were socially advantaged.^26^ In addition, earlier information, such as demographic information during childhood, can be systematically and retrospectively collected, which is referred to as the ‘life grid’ method.^27^ One may speculate potentially poor quality of recalled information on account of potential bias; however, this ‘life grid’ method ensures accuracy of recalled information by using a systematically reconstructed time line specific to the individual participants, providing a time reference to the information of interest. Accuracy of 80% to 90% in childhood information, such as father’s occupation and address, and 100% accuracy in house facilities, were reported when information recalled through the life grid method, collected from a group of older adults, was examined against the information collected 50 years previously.

Missing information can be ignored if researchers decide to use available cases only. However this approach is likely to yield bias unless the distribution of missing cases is completely at random.^28^ Weights to account for missing data can be applied to adjust estimates, as can the application of the full information maximum likelihood method. Multiple imputation by chained equations is also extremely robust at coping with various types of variables and complex datasets. However, it is recommended that distribution of the data, along with missingness patterns, be taken into account in the missing data assumptions. It is best to include all related variables in the imputation model; however, variables with a high rate of non-response can be removed from the analyses to ascertain efficiency.

### Determining causality

As shown in a study by Sacker et al examining the effect of breastfeeding on social mobility,^22^ assigning propensity scores is useful in ascertaining unbiased effects of exposures on outcomes.^29^ This requires the inclusion of all possible confounders in the model for calculation; however, it is impossible to measure all of the possible confounders. Using an instrumental variable, which directly relates to exposures only and is not correlated with confounders, can remedy this problem. Mendelian randomization can be applied to this context, since a candidate gene only relates to a certain phenotype and does not relate to confounders.^30^ However, this approach is quite new, and finding a suitable instrumental variable remains a considerable challenge.

Measurement errors must be reduced to accurately estimate the effect of exposures on outcomes.^29^ Using poorly measured variables with information collected at sequential time points (eg time 1, time 2, and time 3) is likely to yield a poor model fit. In the case of structural equation modeling, including error terms in the model would improve the model fit.

Similarly, the measure of socioeconomic position cannot be treated lightly, especially when health inequalities are assessed over the life course. Bartley^2^ placed social class, status, and material assets, such as income, housing tenure, car, or household amenities, under the umbrella of socioeconomic position. As a reflection of social structure, social class, which is used interchangeably with occupational position, is viewed as sharing large properties with employment conditions and relations, creating a distinct boundary between occupations. The latest occupational position schema—the National Statistics Socio-Economic Classification (NS-SEC)—classifies each occupation into one of seven categories according to timing of remuneration, the presence of pay increments, the possibility of exercising autonomy at work (ie working hours, task design, planning work), and opportunities for promotion.^2^ Being a theoretically derived measure, NS-SEC can address disproportionate poor health in a given occupation with respect to employment relations and conditions.

In contrast, Bartley views social status as a reflection of prestige.^2^ Distinct from social position, social status associated with prestige creates a hierarchal order among people. Income and education can be treated as such measures within this context; however, Bartley discouraged the common practice of combining these two measures into one. She emphasized that this practice does not allow researchers to disentangle the mechanisms of health inequalities, giving an example of two people with different income and health outcomes despite having the same educational background. Since income and education are thought to act as resources as well as sources of prestige,^31^ each measure indicating social position or status is likely to tap into other related factors.

Given this level of complexity, it is advisable to disentangle the social context of each socioeconomic measure to properly model the association between socioeconomic position and health across the life course. Even the effect of education on health may not bear the same magnitude across countries.^32^ Why is health in some individuals poorer than others’, despite similar income, occupation, or education? Researchers need to use socioeconomic measures that are carefully modeled and designed to indicate social gradients in health accurately.

At the same time, the process leading to development of diseases in later life after exposure to socially determined risk factors is thought to progress via interconnected pathways of material, psychosocial, and behavioural factors.^33^ Even so, the roles of these pathways may differ according to pre-existing health, as the protective effect of acquired education in relation to gestational weight gain is conditional, depending on the state of obesity prior to pregnancy.^34^ Allostatic load, estimated by the collection of multiple biomarkers, has been shown to have an association with socioeconomic position.^35^^–^^38^ However, this association was found to be modified by biological events, such as pregnancy.^39^ The challenge of untangling complex mechanisms of social gradients in health across the life course is still ongoing and will only be resolved by collaborative efforts across disciplines.

### Managing longitudinal studies using the life course approach

There is no doubt that longitudinal data collected from an earlier age, rich in contextual information, can offer valuable information for understanding health inequalities across the life course. However, as stated previously, any longitudinal study is likely to suffer from loss of participants, and so great attention is required in communicating with participants.

Participants from a birth cohort study who stayed in the study were interviewed in a qualitative survey.^40^ They identified fulfilment and understanding the importance of the cohort study as the main reasons for their continuing participation. They appeared to have understood that their participation in the study was invaluable. For some participants, the study became ‘a part of their life.’ On the other hand, participation in this cohort study was affected by loss of contact due to frequent moves by the participants. Additionally, study participation was negatively affected by the types of the questions (ie those asking for personal information) and the mode of data collection (ie telephone interview). It is important to reflect feedback from the study participants onto future research designs to ensure continuous cooperation from participants.

British birth cohort data in the public domain (ie open access) are accompanied by detailed documentation. These documents can aid researchers in navigating through the datasets and selecting appropriate study variables as well as in interpreting findings. It is logical that open access data accompanied by detailed documents are likely to prompt researchers to use such data, generating substantial research output and increasing visibility of the studies. Three British birth cohort studies, which are publically available for academic use, are continuously funded by various research councils.^16^^–^^19^ Such financial support may owe something to high visibility achieved through a large volume of research outputs.

## CONCLUSIONS

The application of the life course approach to social epidemiology has advanced the systematic conceptualization of social inequalities in population health. Longitudinal data with rich contextual information collected repeatedly and an advanced statistical approach have been vital in helping obtain research evidence. However, many methodological problems and unanswered questions remain to be addressed, the resolution of which will help researchers refine the theoretical framework of the life course approach. Altogether, researchers will be able to use longitudinal data collected using the life course approach to offer practical advice for reducing health inequalities experienced over the course of one’s life. Collaboration among individual disciplines is needed now more than ever to achieve equality in population health.

## ONLINE ONLY MATERIAL

Abstract in Japanese.

## References

[1] Elder GH Jr, Johnson MK, Crosnoe R. The emergence and development of life course theory. In: Mortimer JT, Shanahan MJ, editors. Handbook of Life Course. New York: Springer; 2003. p. 3–19.

[2] Bartley M. Health inequality: An introduction to theories, concepts and methods. Cambridge: Polity Press; 2004.

[3] Brunner E, Marmot M. Social organisation, stress, and health. In: Marmot M, Wilkinson RG, editors. Social Determinants of Health. Oxford: Oxford Press; 2006. p. 6–30.

[4] Wadsworth M, Butterworth S. Early Life. In: Marmot M, Wilkinson RG, editors. Social Determinants of Health. Oxford: Oxford Press; 2006. p. 31–53.

[5] Kuh D, Ben-Shlomo Y. Introduction. In: Kuh D, Ben-Shlomo Y, editors. A life course approach to chronic disease epidemiology. Oxford: Oxford Press; 2004. p. 3–14.

[6] Lynch J, Smith GD A life course approach to chronic disease epidemiology. Annu Rev Public Health. 2005;26:1–35 10.1146/annurev.publhealth.26.021304.144505 15760279

[7] Ben-Shlomo Y, Kuh D A life course approach to chronic disease epidemiology: conceptual models, empirical challenges and interdisciplinary perspectives. Int J Epidemiol. 2002;31:285–93 10.1093/ije/31.2.285 11980781

[8] Wadsworth M, Maughan B, Pickles A. Introduction: development and progression of life course ideas in epidemiology. In: Pickles A, Maughan B, Wadsworth M, editors. Epidemiological Methods in Life Course Research. Oxford: Oxford Press; 2007. p. 1–25.

[9] Hallqvist J, Lynch J, Bartley M, Lang T, Blane D Can we disentangle life course processes of accumulation, critical period and social mobility? An analysis of disadvantaged socio-economic positions and myocardial infarction in the Stockholm Heart Epidemiology Program. Soc Sci Med. 2004;58:1555–62 10.1016/S0277-9536(03)00344-7 14759698

[10] Blane D. The life course, the social gradient, and health. In: Marmot M, Wilkinson RG, editors. Social Determinants of Health. Oxford: Oxford Press; 2006. p. 54–77.

[11] Ploubidis GB, Destavola BL, Grundy E Health differentials in the older population of England: an empirical comparison of the materialist, lifestyle and psychosocial hypotheses. BMC Public Health. 2011;11:390 10.1186/1471-2458-11-390 21612643 PMC3128018

[12] Schoon I, Sacker A, Bartley M Socio-economic adversity and psychosocial adjustment: a developmental-contextual perspective. Soc Sci Med. 2003;57:1001–15 10.1016/S0277-9536(02)00475-6 12878101

[13] Ploubidis GB, Benova L, Grundy E, et al. Lifelong Socio Economic Position and biomarkers of later life health: A formal comparison of the critical period, accumulation and chans of risk hypotheses. European population conference. Stockholm, Sweden. 2012. p. 1–22.

[14] Murray ET, Mishra GD, Kuh D, Guralnik J, Blacks S, Hardy R Life course models of socioeconomic position and cardiovascular risk factors: 1946 birth cohort. Ann Epidemiol. 2011;21:589–97 10.1016/j.annepidem.2011.04.005 21737047 PMC3226834

[15] Mishra GD, Chiesa F, Goodman A, De Stavola B, Koupil I Socio-economic position over the life course and all-cause, and circulatory diseases mortality at age 50–87 years: results from a Swedish birth cohort. Eur J Epidemiol. 2013;28:139–47 10.1007/s10654-013-9777-z 23435736

[16] Wadsworth M, Kuh D, Richards M, Hardy R Cohort profile: The 1946 National Birth Cohort (MRC National Survey of Health and Development). Int J Epidemiol. 2006;35:49–54 10.1093/ije/dyi201 16204333

[17] Power C, Elliott J Cohort profile: 1958 British birth cohort (National Child Development Study). Int J Epidemiol. 2006;35:34–41 10.1093/ije/dyi183 16155052

[18] Elliott J, Shepherd P Cohort profile: 1970 British Birth Cohort (BCS70). Int J Epidemiol. 2006;35:836–43 10.1093/ije/dyl174 16931528

[19] Connelly R, Platt L Cohort profile: UK Millennium Cohort Study (MCS). Int J Epidemiol. 2014 Feb 17; doi:10.1093/ije/dyu001 10.1093/ije/dyu001 24550246

[20] Lacey RE, Bartley M, Pikhart H, Stafford M, Cable N, Coleman L Parental separation and adult psychological distress: evidence for the ‘reduced effect’ hypothesis? Longitudinal and Life Course Studies: International Journal. 2012;3:359–68 10.14301/llcs.v3i3.195

[21] Cable N, Bartley M, McMunn A, Kelly Y Gender differences in the effect of breastfeeding on adult psychological well-being. Eur J Public Health. 2012;22:653–8 10.1093/eurpub/ckr135 22021373

[22] Sacker A, Kelly Y, Iacovou M, Cable N, Bartley M Breast feeding and intergenerational social mobility: what are the mechanisms? Arch Dis Child. 2013;98:666–71 10.1136/archdischild-2012-303199 23798701 PMC3756446

[23] Centre for Longtifudinal Study. Millennium Cohort Study First, Second, Third ad Fourth Surveys A Guide to the Datasets (Eighth Edition). In: Hansen K, editor. London: Centre for Longitudinal Study; 2014. p. 1–107.

[24] Hockley C, Quigley MA, Hughes G, Calderwood L, Joshi H, Davidson LL Linking Millennium Cohort data to birth registration and hospital episode records. Paediatr Perinat Epidemiol. 2008;22:99–109 18173788 10.1111/j.1365-3016.2007.00902.x

[25] Quigley MA, Hockley C, Davidson LL Agreement between hospital records and maternal recall of mode of delivery: evidence from 12 391 deliveries in the UK Millennium Cohort Study. BJOG. 2007;114:195–200 10.1111/j.1471-0528.2006.01203.x 17166217

[26] Tate AR, Dezateux C, Cole TJ, Davidson L; Millennium Cohort Study Health Group Factors affecting a mother’s recall of her baby’s birth weight. Int J Epidemiol. 2005;34:688–95 10.1093/ije/dyi029 15737964

[27] Berney LR, Blane DB Collecting retrospective data: accuracy of recall after 50 years judged against historical records. Soc Sci Med. 1997;45:1519–25 10.1016/S0277-9536(97)00088-9 9351141

[28] Clarke P, Hardy R. Methods for handling missing data. In: Pickles A, Maughan B, Wadsworth M, editors. Epidemiological Methods in Life Course Research. Oxford: Oxford University Press; 2007. p. 157–79.

[29] Pickles A, De Stavola B. An overview of models and methods for life course analysis. In: Pickles A, Maughan B, Wadsworth M, editors. Epidemiological methods in life course research. Oxford: Oxford University Press; 2007. p. 181–220.

[30] Didelez V, Sheehan N Mendelian randomization as an instrumental variable approach to causal inference. Stat Methods Med Res. 2007;16:309–30 10.1177/0962280206077743 17715159

[31] Krieger N, Williams DR, Moss NE Measuring social class in US public health research: concepts, methodologies, and guidelines. Annu Rev Public Health. 1997;18:341–78 10.1146/annurev.publhealth.18.1.341 9143723

[32] Howe LD, Galobardes B, Matijasevich A, Gordon D, Johnson D, Onwujekwe O, Measuring socio-economic position for epidemiological studies in low- and middle-income countries: a methods of measurement in epidemiology paper. Int J Epidemiol. 2012;41:871–86 10.1093/ije/dys037 22438428 PMC3396323

[33] Blane D, Kelly-Irving M, d’Errico A, Bartley M, Montgomery S Social-biological transitions: how does the social become biological? Longitudinal and Life Course Studies: International Journal. 2013;4:136–46 10.14301/llcs.v4i2.236

[34] Holowko N, Mishra G, Koupil I Social inequality in excessive gestational weight gain. Int J Obes. 2014;38:91–6 10.1038/ijo.2013.62 23711774

[35] Gustafsson PE, Janlert U, Theorell T, Westerlund H, Hammarström A Socioeconomic status over the life course and allostatic load in adulthood: results from the Northern Swedish Cohort. J Epidemiol Community Health. 2011;65:986–92 10.1136/jech.2010.108332 20974835

[36] Robertson T, Popham F, Benzeval M Socioeconomic position across the lifecourse & allostatic load: data from the West of Scotland Twenty-07 cohort study. BMC Public Health. 2014;14:184 10.1186/1471-2458-14-184 24555560 PMC3942053

[37] Rainisch BK, Upchurch DM Sociodemographic correlates of allostatic load among a national sample of adolescents: findings from the National Health and Nutrition Examination Survey, 1999–2008. J Adolesc Health. 2013;53:506–11 10.1016/j.jadohealth.2013.04.020 23763967 PMC3783611

[38] Steptoe A, Feldman PJ, Kunz S, Owen N, Willemsen G, Marmot M Stress responsivity and socioeconomic status: a mechanism for increased cardiovascular disease risk? Eur Heart J. 2002;23:1757–63 10.1053/euhj.2001.3233 12419295

[39] Morrison S, Shenassa ED, Mendola P, Wu T, Schoendorf K Allostatic load may not be associated with chronic stress in pregnant women, NHANES 1999–2006. Ann Epidemiol. 2013;23:294–7 10.1016/j.annepidem.2013.03.006 23621995 PMC4132932

[40] Parsons S. Understanding participation: Being a part of the National Child Development Study from birth to age 50. London: Centre for Longitudinal Studies; 2010. p. 1–29.

