# Supplementary material for: Life Course Approach in Social Epidemiology: An Overview, Application and Future Implications
Source: J Epidemiol. 2014 Sep 5;24(5):347–52. doi: 10.2188/jea.JE20140045 (PMC4150004; doi:10.2188/jea.JE20140045)
Supplement: Abstract in Japanese. [file je-24-347-s001.pdf]

社会疫学におけるライフコースアプローチ: 概要, 適用及び将来に向けての展望

Noriko Cable, Research Department of Epidemiology and Public Health, University College London

ライフコースアプローチの社会疫学の分野への適用は、社会格差が国民の健康にどう影響を及ぼすかを理論的に研究するために役立っている。繰り返し収集された文脈的要因情報に富む縦断データや高度な統計手法によりライフコースアプローチによる社会格差の理論的な解明の困難さが軽減されていると言えよう。
本総説は、疫学におけるライフコースアプローチの概要、適用、及び将来に向けての課題を述べたものである。疫学研究者がライフコースアプローチによる健康格差縮小への確たる指標を打ち出すには 、理論的に裏付けられた社会経済地位測定因子も含め、体系的な考えに基づいた研究手法の適用、ならびに研究分野の枠を越えた各研究者の協力が望まれる。

キーワード：ライフコースアプローチ、社会格差、社会疫学
